# Subtype-specific prognostic impact of TNIK in medulloblastoma

**DOI:** 10.1007/s11060-026-05554-y

**Published:** 2026-05-08

**Authors:** Franz-Leonard Klaus, Theoni Maragkou, Claire Delbridge, Charles G. Eberhardt, Ekkehard Hewer, Antonia Gocke, Ramin Radpour, Christian Mawrin, Carolin Mogler, Julia E. Neumann, Stefan Forster

**Affiliations:** 1 Institute of Pathology, Technical University Munich, Munich, Germany; 2 Institute of Tissue Medicine and Pathology, University of Bern, Bern, Switzerland; 3 Department of Pathology, School of Medicine, Johns Hopkins University, Baltimore, MD USA; 4 Department of Laboratory Medicine and Pathology, Institute of Pathology, Lausanne University Hospital and University of Lausanne, Lausanne, Switzerland; 5 Center for Molecular Neurobiology Hamburg (ZMNH), University Medical Center Hamburg-Eppendorf (UKE), Hamburg, Germany; 6 Center for Diagnostics, Section Mass Spectrometry and Proteomics, University Medical Center Hamburg-Eppendorf, Hamburg, Germany; 7 Tumor Immunology, Department for BioMedical Research (DBMR), University of Bern, Bern, Switzerland; 8 Department of Medical Oncology, Inselspital, Bern University Hospital, University of Bern, Bern, Switzerland; 9 Department of Neuropathology, Otto-von-Guericke-University, Magdeburg, Germany; 10 Institute of Neuropathology, University Medical Center Hamburg-Eppendorf (UKE), Hamburg, Germany

**Keywords:** Medulloblastoma, TNIK, Prognostic biomarker, Molecular subgroups, Wnt signaling

## Abstract

**Purpose:**

Medulloblastoma (MB) is the most common malignant pediatric brain tumor and comprises molecularly and clinically distinct subgroups with highly variable outcomes. While survival rates exceed 80% in some subgroups, others remain associated with substantially poorer prognosis and increased risk of relapse. Current treatment includes surgery, radiation, and chemotherapy, which can result in significant long-term treatment-related morbidity. These challenges highlight the need to identify novel biomarkers and potential therapeutic targets to improve risk stratification and enable more tailored treatment approaches. The Traf2- and Nck-interacting kinase (TNIK) is a regulator of Wnt/β-catenin signaling and has been implicated in the progression of several cancers, but its role in MB remains unclear.

**Methods:**

TNIK expression was evaluated using publicly available MB transcriptomic datasets, mass spectrometry based proteomic data, and immunohistochemical analysis of a MB tissue microarray. Associations between TNIK expression, molecular subgroups, overall survival, and established prognostic markers were assessed.

**Results:**

TNIK expression was significantly enriched in Group 3 and Group 4 MBs compared to WNT and SHH subgroups. Survival analyses demonstrated distinct, subgroup-specific prognostic effects: high TNIK expression predicted inferior overall survival in Group 4, while in SHH tumors high TNIK expression levels were associated with improved outcome. These observations were supported by proteomic profiling.

**Conclusion:**

These findings indicate a subgroup-specific role for TNIK in MB biology and suggest its potential utility as a prognostic biomarker, particularly in Group 4 MBs.

**Supplementary Information:**

The online version contains supplementary material available at 10.1007/s11060-026-05554-y.

## Introduction

Medulloblastoma (MB) is the most common malignant pediatric brain tumor and is characterized by its aggressive growth pattern with a high risk for cerebrospinal fluid dissemination. Based on its molecular alterations, MBs are classified into four distinct subgroups, namely Wnt-activated, SHH-activated, Group 3 and Group 4 MBs [1, 2]. Wnt-activated MBs account for only ~10% of all cases and are characterized by activating mutations in *CTNNB1*, resulting in a constitutive Wnt-pathway activation [3, 4]. SHH-activated MBs are characterized by aberrant activation of the Sonic Hedgehog signaling cascade, most commonly through mutations in *PTCH1*, *SMO*, or *SUFU* [5]. In contrast, Group 3 MBs frequently harbor *MYC* amplifications and display a high propensity for aggressive behavior and metastasis [4]. Group 4, the most common subgroup, remains the least well biologically characterized [1, 4]. While Wnt-activated MBs are often associated with a better prognosis, Group 3 and Group 4 MBs, as well as SHH MBs harboring mutations in *TP53*, show the least favorable outcomes [6, 7]. Notably, recent integrative genomic and epigenomic studies have demonstrated that each of the four subgroups comprises multiple biologically and clinically distinct subtypes with divergent prognostic courses. These subclassifications, defined by transcriptomic and DNA methylation profiling, have revealed substantial heterogeneity within subgroups, including both favorable- and high-risk subtypes within SHH, Group 3, and Group 4 MBs. This refined molecular stratification has improved understanding of MB biology and is increasingly relevant for prognostic assessment and risk-adapted therapeutic approaches [8–10]. MB treatment often involves a combination of surgery, radiation, and chemotherapy, but the debilitating side effects frequently limit its effectiveness, highlighting the need for more targeted therapies with fewer side effects [11]. Because aberrant Wnt/β-catenin signaling is a central feature of MB biology, kinases that regulate this pathway may represent attractive therapeutic targets.

The Traf2- and Nck-interacting kinase (TNIK) is a serine/threonine kinase, acting as a key effector of Wnt/β-catenin signaling by interacting with β-catenin and TCF4, thereby enhancing Wnt target gene expression [12]. It plays a crucial role in cancer stem cell (CSC) maintenance, tumor progression, and therapeutic resistance [13]. In colorectal, gastric, pancreatic, and hepatocellular carcinomas, high TNIK expression correlates with poor prognosis by promoting tumor growth, invasion, and chemoresistance [14–18]. TNIK overexpression is also linked to colorectal and breast cancer stemness, where it supports self-renewal and oncogenesis [15, 19]. In glioblastoma, low TNIK expression levels have been associated with worse prognosis and activation of the Hippo pathway, suggesting a potential tumor-suppressive role in this context [20]. In MB pathogenesis and prognosis, the role of TNIK remains largely unexplored, leaving its potential impact on tumor biology and patient outcomes unclear.

This study aims to assess TNIK expression across MB subtypes, evaluate its prognostic relevance, and explore subtype-specific biological functions through gene set enrichment analyses. Building on its established role in Wnt/β-catenin signaling, CSC maintenance, and therapy resistance in multiple malignancies, we sought to determine whether TNIK exhibits subtype-dependent biological and clinical associations in MB that may provide a rationale for future functional and therapeutic investigations.

## Materials & methods

### Public dataset analysis

Data from three independent MB databases (*GSE85217*, *GSE21140* and *GSE37418)* were extracted. TNIK mRNA expression was analyzed in relation to overall survival using the *GSE85217* dataset and patients were stratified into TNIK-high and TNIK-low groups within the individual subgroups. The optimal cut-off for TNIK expression was determined using X-tile software (Yale University, version 3.6.1) [21], which identifies outcome-based thresholds by assessing all possible cut-off points and selecting the value that yields the most significant separation between survival groups using the log-rank test. Cut-offs were calculated independently for each molecular subgroup using TNIK mRNA expression as a continuous variable and overall survival as the clinical endpoint. Kaplan-Meier survival analysis, log-rank testing and visualizations were performed in R (v.4.2.0) using the packages survival, survminer, ggsurvfit, ggplot2 and ggbeeswarm. Gene set enrichment analyses were conducted with fgsea, using gene sets retrieved via msigdbr, and visualized with clusterProfiler. Gene annotation was performed with org.Hs.eg.db, hugene11sttranscriptcluster.db, and biomaRt.

A detailed description of materials and methods is provided in the Supplemental Data.

## Results

### TNIK expression is increased in Group 3 and Group 4 Medulloblastomas

**Fig. 1:**
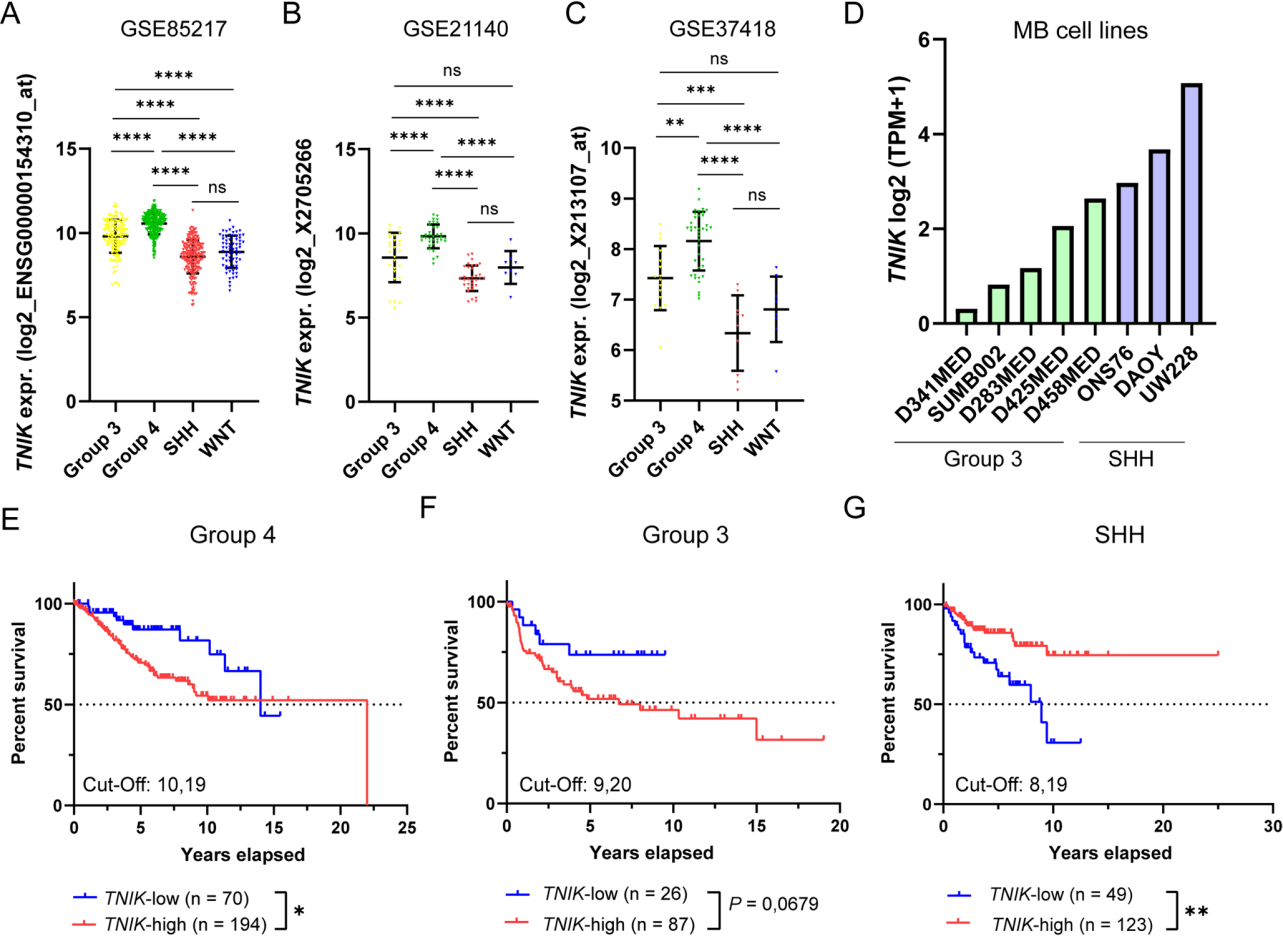
TNIK is a prognostic biomarker in medulloblastoma. TNIK expression was assessed across MB subtypes (Wnt, SHH, Group3 and Group 4), data extracted from three independent datasets *GSE85217* (**A**), *GSE21140* (**B**) and *GSE37418* (**C**). TNIK expression in MB cell lines from SHH (*n* = 3) and Group 3 (*n* = 5) molecular backgrounds; data were extracted from the cancer cell line encyclopedia (CCLE) (**D**). Kaplan-Meier survival curves of TNIK-high and TNIK-low MBs in Group 4 (**E**), Group 3 (**F**) and SHH (**G**) subtypes. Statistics: one-way ANOVA with post-hoc Tukey HSD test (**A**-**C**), log-rank test (**E**-**G**); *, *P* < 0.05; **, *P* < 0.01; ***, *P* < 0.001; ****, *P* < 0.0001; Data are shown as mean with standard deviation (SD)

To evaluate TNIK expression in MB, we first analyzed gene expression data from the *GSE85217* dataset, which includes 763 primary MBs representing various molecular subgroups [8]. We found that TNIK expression was significantly increased in Group 3 and Group 4 MBs compared to WNT and SHH subgroups, with the highest expression levels observed in Group 4 MBs (Fig. 1A). These findings were validated in two additional independent MB datasets, *GSE21140* and *GSE37418* (Fig. 1B and C). To further define subgroup-specific expression patterns, we analyzed TNIK expression across molecular subtypes within each MB subgroup. This analysis revealed substantial heterogeneity at the subtype level. TNIK expression was highest in Group 3β and Group 4β tumors within Group 3 and Group 4 MB, respectively, as well as in SHHβ and SHHγ subtypes. Within the WNT subgroup, TNIK expression was significantly higher in WNTα compared to WNTβ tumors (Suppl. Figure 1A). These findings highlight marked intra-subgroup variability in TNIK expression. Additionally, heterogeneous TNIK expression was confirmed in eight MB cell lines from different molecular subgroups (*n* = 5 Group 3 MBs; *n* = 3 SHH MBs) (Fig. 1D) [22]. Using an X-tile-based cut-off separation in relation to overall survival (OS) of MB patients [21], we found that high TNIK mRNA expression was significantly associated with poorer OS in Group 4 MBs alone, as well as in the combined Group 3 and Group 4 cohort (Fig. 1E and Suppl. Figure 1B). A similar, though non-significant, trend toward reduced OS was observed in Group 3 MBs with high TNIK expression (Fig. 1F). Conversely, in SHH MBs, high TNIK expression correlated with improved OS, while patients with low TNIK expression exhibited significantly worse survival outcomes (Fig. 1G). Due to the consistently favorable prognosis and limited number of events in the WNT subgroup, stratification according to TNIK expression was not feasible for this cohort (Suppl. Figure 1C). Descriptive inspection of the few cases with events (WNTα: 1/49; WNTβ: 2/21) did not reveal elevated or outlier TNIK expression compared to subgroup means (WNTα: 8.33 vs. 9.21; WNTβ: 7.66 vs. 8.05). Furthermore, stratification of MB subtypes into TNIK-high and TNIK-low groups using the X-tile derived cut-offs did not reveal any statistically significant differences, except within the Group 4α, where high *TNIK* expression was associated with significantly inferior survival (Suppl. Figure 1D–F).

We further examined clinical and histopathological parameters such as patient age, metastatic status, and tumor histology between TNIK-high and TNIK-low MB patients within each molecular subgroup. In Group 4 MBs, TNIK-low patients were significantly younger than those with high TNIK expression. In Group 3 MBs, metastasis was more frequently observed in TNIK-low patients. Within the SHH subgroup, desmoplastic histology was more prevalent in TNIK-high tumors, whereas large cell/anaplastic (LCA) features were more commonly found in TNIK-low tumors, suggesting that TNIK-high SHH MBs may represent a more differentiated phenotype (Table 1).

**Table 1:** Comparison of clinical and histopathologic parameters between TNIK-low and TNIK-high MB subgroups

**SHH**	**TNIK-low** **(** ***n*** ** = 68)**	**TNIK-high** **(** ***n*** ** = 155)**	***P*** **-value**
Sex			
Male	37 (57.8%)	91 (62.3%)	ns
Female	27 (42.2%)	55 (37.7%)	ns
Mean Age	15.8	12.6	ns
Metastasis	7 (14.6%)	19 (17%)	ns
No Metastasis	41 (85.4%)	93 (83%)	
Histologic growth pattern			
Classic	24 (46.2%)	54 (41.9%)	ns
Desmoplastic	13 (25%)	60 (46.5%)	0.0078
LCA	13 (25%)	7 (5.4%)	0.0004
MBEN	2 (3.8%)	8 (6.2%)	ns
**Group 3**	**TNIK-low** **(** ***n*** **= 31)**	**TNIK-high** **(** ***n*** **= 113)**	***P*** **-value**
Sex			
Male	19 (67.9%)	80 (73.4%)	ns
Female	9 (32.1%)	29 (26.6%)	ns
Mean Age	6	7.1	ns
Metastasis	17 (70.8%)	26 (30.6%)	0.0007
No Metastasis	7 (29.2%)	59 (69.4%)	
Histologic growth pattern			
Classic	19 (82.6%)	49 (61.3%)	0.0798
Desmoplastic	1 (4.3%)	7 (8.8%)	ns
LCA	3 (13%)	22 (27.5%)	ns
MBEN	0 (0%)	2 (2.5%)	ns
**Group 4**	**TNIK-low** **(** ***n*** **= 87)**	**TNIK-high** **(** ***n*** **= 239)**	***P*** **-value**
Sex			
Male	57 (69.5%)	159 (70.4%)	ns
Female	25 (30.5%)	67 (29.6%)	ns
Mean Age	8.3	9.5	0.047
Metastasis	29 (45.3%)	72 (37.7%)	ns
No Metastasis	35 (54.7%)	119 (62.3%)	
Histologic growth pattern			
Classic	47 (71.2%)	154 (82.8%)	0.0509
Desmoplastic	8 (12.1%)	15 (8.1%)	ns
LCA	8 (12.1%)	14 (7.5%)	ns
MBEN	3 (4.5%)	3 (1.6%)	ns

Overall, our data suggest that TNIK expression is significantly elevated in Group 3 and Group 4 MBs and that high TNIK levels are associated with worse OS in these groups. In contrast, high TNIK expression in SHH MBs correlates with a better OS, suggesting subgroup-specific prognostic implications.

### TNIK expression correlates with subgroup-dependent cytogenetic alterations in medulloblastoma

**Fig. 2:**
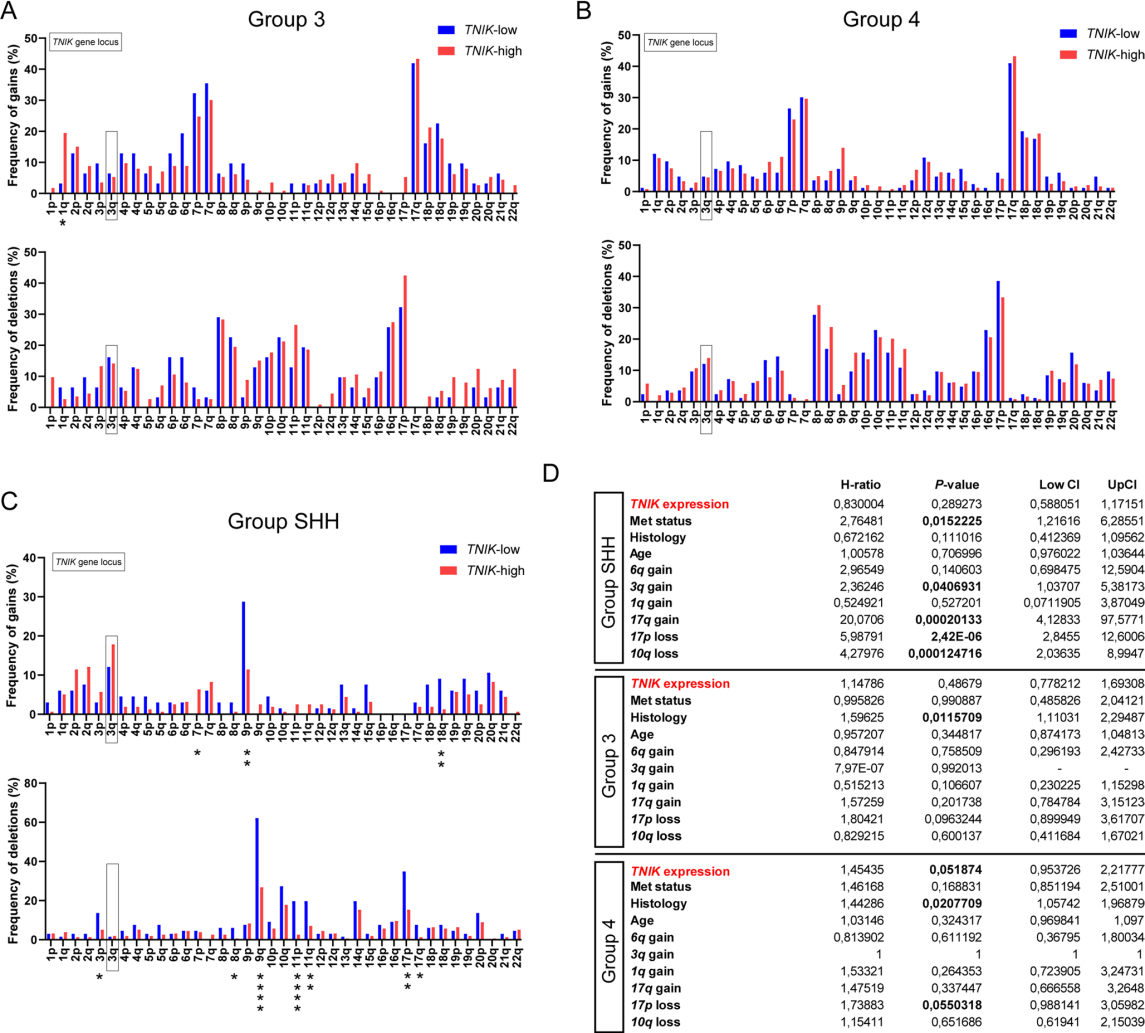
Correlation of TNIK expression and cytogenetic profiles across molecular subgroups in medulloblastoma. The cytogenetic backgrounds (gains and deletions of specific chromosomal regions) were compared between TNIK-low and TNIK-high MB patients across Group 3 (**A**) Group 4 (**B**) and SHH (**C**) subgroups. Role of TNIK as an independent prognostic marker in medulloblastoma (**D**). Statistics: Fisher’s exact test (**A**-**C**), multiple Cox regression (**D**); *, *P* < 0.05; **, *P* < 0.01; ***, *P* < 0.001; ****, *P* < 0.0001; Data are shown as mean with SD

TNIK is located on chromosome *3q*, a chromosomal region where gains have been associated with poor outcomes in MB [23]. To assess whether TNIK expression is influenced by alterations of the chromosomal region *3q*, we compared the frequency of *3q* gains and deletions between TNIK-high and TNIK-low MBs. No significant differences were observed in the rates of *3q* gains or losses between the two groups, implying that other regulatory mechanisms might be responsible for elevated TNIK expression in MB (Fig. 2A–C).

Since specific cytogenetic alterations are linked to adverse prognosis in MB, we further analyzed the given cytogenetic profiles of TNIK-high or -low tumors across different molecular subgroups [23–25]. In Group 3 MBs, gain of chromosome *1q* was significantly more frequent in TNIK-high tumors compared to TNIK-low cases, while no other notable differences were detected (Fig. 2A). In SHH MBs, gain of chromosome *7p* was more common in TNIK-high tumors, whereas gains of *9p* and *18q* occurred more frequently in TNIK-low tumors. Additionally, deletions of *3p*, *8q*, *9q*, *11p*, *11q*, *17p*, and *17q* were significantly more prevalent in TNIK-low SHH tumors compared to those with high TNIK expression. In contrast, no significant differences in cytogenetic alterations were observed between TNIK-high and -low tumors in Group 4 MBs (Fig. 2B and C).

To further assess whether TNIK functions as an independent prognostic biomarker, we conducted multivariate analyses that included age, metastatic status, histological subtype, and key cytogenetic alterations (e.g., *17p* deletion, *17q* gain, and *6q* gain) [23]. TNIK as a continuous variable was not identified as an independent prognostic factor in Group 3 or SHH MBs. However, in Group 4 MBs, there was a strong trend suggesting that TNIK may serve as an independent prognostic marker in this subgroup (Fig. 2D).

In summary, TNIK expression does not significantly correlate with gains or deletions of the *3q* chromosomal region but shows distinct cytogenetic associations across the molecular subgroups. Moreover, TNIK exhibited a borderline independent prognostic effect in multivariable Cox regression analysis in Group 4 MBs.

### TNIK is heterogeneously expressed at proteomic protein levels in MB subtypes and correlates with patients’ prognosis

**Fig. 3:**
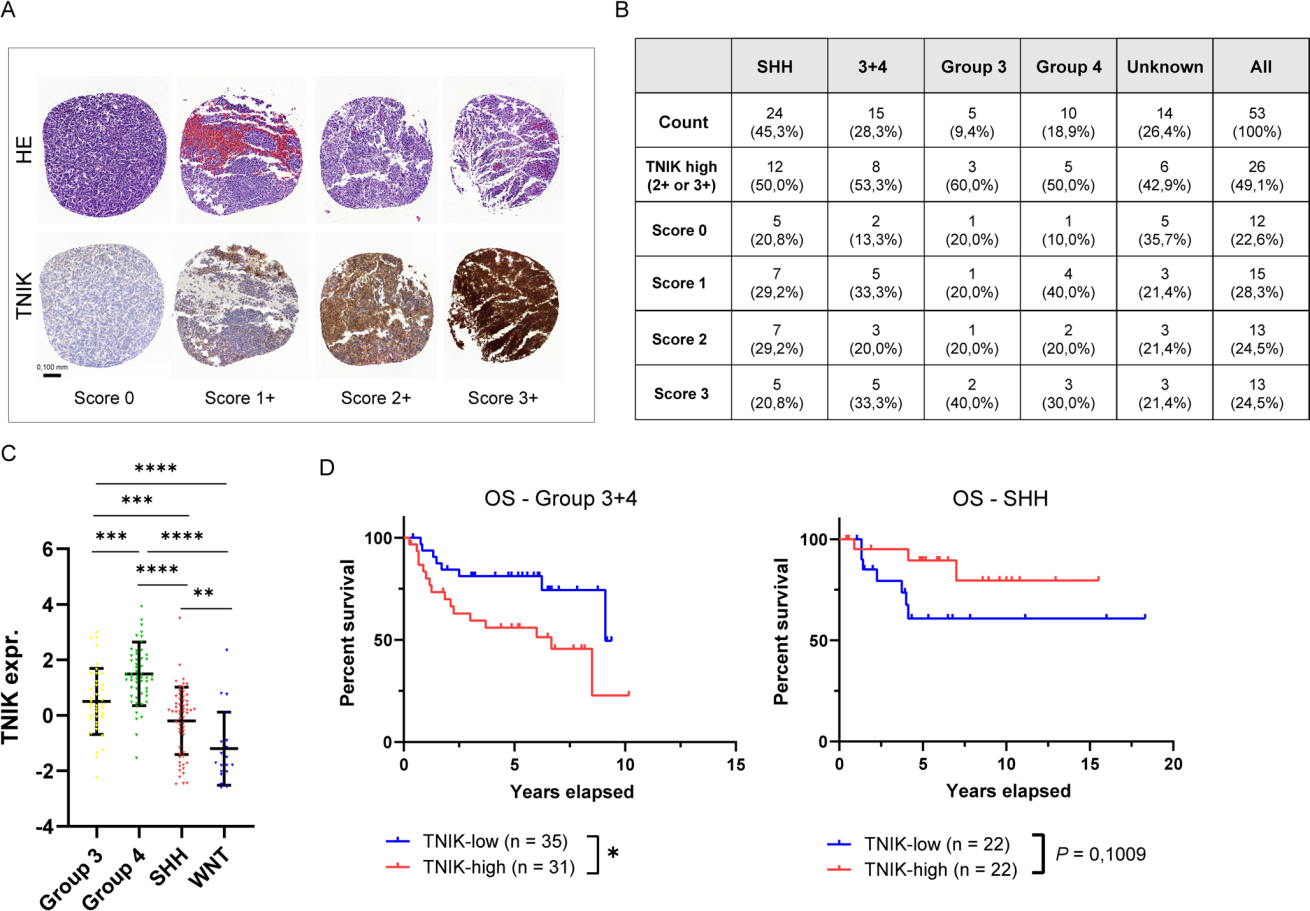
TNIK protein expression levels in a medulloblastoma tissue microarray and mass spectrometry dataset. Immunohistochemistry staining for TNIK was performed on a TMA containing 53 primary MB specimens and TNIK expression was scored according to its intensity from 0 to 3+ (**A** and **B**). Mass spectrometry-based TNIK proteomic profiles across MB subtypes (**C**). Overall survival (OS) of Group 3 + 4 combined MBs and SHH-MBs stratified into TNIK-low and TNIK-high subgroups based on the median mass spectrometry cutoff (**D**). Statistics: one-way ANOVA with post-hoc Tukey HSD test (**C**), log-rank test (**D**); **, *P* < 0.01; ***, *P* < 0.001; ****, *P* < 0.0001; Data are shown as mean with SD

To assess TNIK protein expression in MBs, we additionally analyzed a tissue microarray (TMA) containing 53 primary MB specimens from different molecular subgroups using immunohistochemistry staining (Suppl. Figure 1G). TNIK expression was heterogeneous among MB patients, ranging from no expression (*n* = 12; 22.6%) to low (*n* = 15; 28.3%), moderate (*n* = 13; 24.5%), and high (*n* = 13; 24.5%) expression levels (Fig. 3A and B). In contrast, only low to moderate TNIK expression was found in normal cerebellum tissue sections (Suppl. Figure 1H) [26, 27].

In our MB TMA, strong TNIK protein expression (score 3+) was observed in Group 3 (2/5, 40.0%), Group 4 (3/10, 30.0%), and SHH MBs (5/24, 20.8%) (Fig. 3B). When combining Group 3 and Group 4 tumors, strong TNIK expression was observed in 5/15 cases (33.3%); however, compared with SHH MBs, this did not reach statistical significance (Fisher’s exact test, *P* = 0.46) (Fig. 3B). In addition, we analyzed mass spectrometry–based proteomic data and correlated TNIK protein levels with OS across MB subtypes. Consistent with our gene expression and TMA data, the highest TNIK protein expression levels were observed in Group 3 and Group 4 MBs (Fig. 3C). In these subgroups, high TNIK expression showed a strong trend toward reduced OS, and combining both cohorts revealed a significant survival difference (Fig. 3D and Suppl. Figure 1I-J). By contrast, in SHH MBs, high TNIK expression was associated with a strong trend toward improved outcomes (Fig. 3D).

### TNIK exerts different biological functions dependent on the molecular subtype

To explore TNIK-related signaling across MB subgroups, we stratified SHH, Group 3, and Group 4 tumors by TNIK gene expression levels (Fig. 1E–G) to identify differentially expressed genes and pathways between TNIK-high and TNIK-low samples. In Group 3 MBs, 117 genes were significantly upregulated, while 57 were downregulated (Suppl. Figure 2A). The top upregulated genes in TNIK-high Group 3 MBs included *RPH3A*, *DCT*, and *ELAVK4*, while *LRP2* was significantly downregulated (Suppl. Figure 2B). Gene set enrichment analyses (GSEA) were performed to assess the impact of TNIK expression on biological processes and signaling pathways. GSEA revealed downregulation of genes associated with cell cycle induction, proliferation, and epithelial-to-mesenchymal transition (EMT), while genes related to the Hedgehog signaling pathway were significantly enriched in TNIK-high Group 3 MBs (Suppl. Figure 2C).

**Fig. 4:**
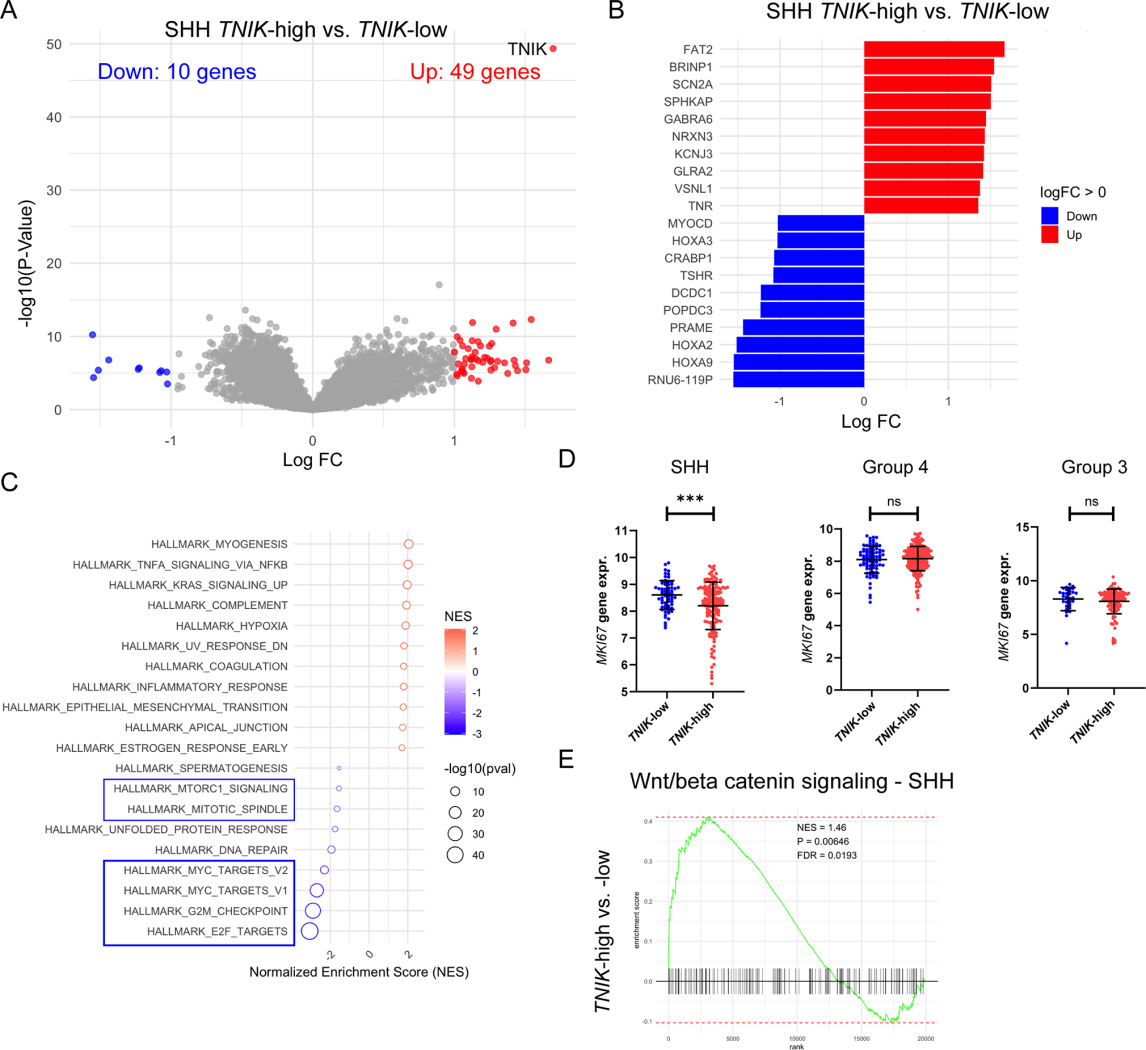
Biological and mechanistic functions of TNIK in medulloblastoma subgroups. Volcano-plot visualizing significantly upregulated and downregulated genes in TNIK-high versus TNIK-low SHH MB patients (**A**). Top ten upregulated and downregulated genes in TNIK-high SHH MBs (**B**). Gene set enrichment analyses (GSEA) of TNIK-high versus TNIK-low SHH MBs (**C**). *MKI67* expression levels across TNIK-high versus TNIK-low tumors from SHH. Group 3 and Group 4 backgrounds (**D**). Enrichment of genes involved in Wnt/β-catenin signaling in TNIK-high versus TNIK-low SHH MB patients (**E**). Statistics: student´s t-test (**D**); ***, *P* < 0.001; Data are shown as mean with SD

In Group 4 MBs, only 8 genes were significantly upregulated in the TNIK-high cohort, with no significantly downregulated genes observed (Suppl. Figure 2D). Among these, *EOMES* was notably upregulated in TNIK-high Group 4 MBs (Suppl. Figure 2E). Similar to Group 3, GSEA in Group 4 MBs showed a significant downregulation of genes related to EMT in TNIK-high tumors (Suppl. Figure 2F). In SHH MBs, 49 genes were significantly upregulated, while 10 were downregulated, with *HOXA2* and *HOXA9* showing strong negative expression values (Fig. 4A and B). As in Group 3, genes involved in cell cycle transition and cell proliferation were significantly downregulated in TNIK-high SHH MBs. However, unlike in Group 3 and Group 4, TNIK-high SHH MBs exhibited enrichment of genes linked to EMT (Fig. 4C). Given the association of TNIK with cell cycle induction and proliferation, we compared *MKI67* expression levels between TNIK-high and -low MBs. *MKI67* expression was significantly higher in TNIK-low SHH MBs, indicating increased proliferation (Fig. 4D). No significant differences in *MKI67* expression levels were detected in Group 3 or Group 4 MBs (Fig. 4D). Since TNIK is known to activate the Wnt/β-catenin signaling pathway [12, 28], we further examined the enrichment of Wnt/β-catenin-dependent genes in TNIK-high versus TNIK-low MBs. Wnt/β-catenin signaling was significantly induced in TNIK-high SHH MBs, whereas no significant changes were observed in Group 3 or Group 4 TNIK-high tumors (Fig. 4E and Suppl. Figure 2G). Overall, our data suggest that TNIK might exert distinct biological functions depending on the molecular subtype of MB.

## Discussion

Using publicly available MB gene expression datasets, a MB tissue microarray and mass spectrometry data of MB patients, we demonstrate that the serine/threonine kinase TNIK is positively expressed in approximately 50% of MB patients, acting as a potential prognostic biomarker with subgroup-specific biological relevance.

While high TNIK expression is associated with improved OS in SHH-activated MBs, the opposite effect was observed in Group 3/4 tumors, where TNIK-high status correlates with worse clinical outcomes. Furthermore, TNIK expression is significantly lower in SHH compared to Group 3/4 tumors, suggesting subtype-specific differences in TNIK regulation. Despite TNIK being located on chromosome *3q*, we did not observe an association between elevated TNIK expression in Group 3/4 MBs and *3q* gains. This suggests that its upregulation in MB is not primarily driven by copy number alterations. Instead, alternative mechanisms may be involved. These may include epigenetic regulation such as TNIK promoter methylation, as recently reported in thyroid cancer [29]. Moreover, the induction of TNIK expression by distinct effector molecules such as BASP1, the METTL3/FAM135B axis, or miR-5590-3p has also been described in colorectal cancer, esophageal cancer or ovarian cancer, respectively [30–32]. Divergent patterns were further reinforced by subtype-restricted associations with specific chromosomal alterations, proliferation markers, and gene set enrichment profiles, indicating that TNIK may play fundamentally different roles in SHH versus non-SHH MBs. Specifically, TNIK*-*high SHH tumors exhibited significantly lower *MKI67* expression, indicating reduced proliferative activity, and showed enrichment for Wnt-signaling pathways in GSEA. This aligns with preclinical studies demonstrating that activation of the canonical Wnt/β-catenin pathway suppresses SHH-driven tumorigenesis by antagonizing the SHH signaling cascade and promoting cellular differentiation [33]. Conversely, TNIK-low SHH tumors were characterized by increased activation of proliferative gene sets such as E2F targets, G2M checkpoint, mitotic spindle, and MYC targets, suggesting a shift toward aberrant cell cycle progression. Additionally, we observed that TNIK-low SHH tumors more frequently harbored deletions of the chromosomal regions *17p* as well as *9q*. Notably, loss of the chromosomal regions *9q* and *17p* (*TP53*) have been associated with poor prognosis in MBs [23, 34]. These findings suggest that high TNIK expression in SHH MBs may reflect a more differentiated, less proliferative phenotype.

In contrast to its apparent tumor-suppressive role in SHH MBs, high TNIK expression was associated with inferior survival in Group 3/4 tumors in cut-off-based Kaplan–Meier analyses. In multivariable models, TNIK was treated as a continuous variable, and the weaker association may reflect potential non-linear or threshold-dependent effects. Notably, TNIK expression was significantly higher in Group 3/4 compared to SHH tumors, and TNIK-high status in this subgroup did not significantly correlate with enrichment of Wnt-related genes in gene set enrichment analysis. TNIK gene expression showed no significant association with *MKI67* expression, suggesting that in Group 3/4 tumors, TNIK may promote tumor progression through mechanisms independent of canonical Wnt-signaling. TNIK expression in Group 3 MBs showed a dissociation between metastatic presentation and survival outcomes. While TNIK-low tumors exhibit higher rates of metastasis at diagnosis, TNIK-high tumors were associated with significantly poorer OS, suggesting that TNIK reflects tumor aggressiveness beyond initial dissemination. Mechanistically, TNIK acts as an effector of Wnt/β-catenin signaling, promoting proliferation, stemness, and therapy resistance, which may drive adverse outcomes independent of metastatic status. This indicates that metastatic potential and long-term tumor behavior may be governed by partially distinct biological programs. Consequently, TNIK may serve as a marker of tumor aggressiveness rather than a direct indicator of metastatic capacity in MBs, specifically in Group 3 MBs.

Since Group 3/4 MBs are largely considered “Wnt-inactive” or “Wnt-naïve,” the absence of canonical Wnt pathway dependence in these tumors may render TNIK’s classical function as a Wnt effector less relevant, potentially shifting its role toward alternative signaling mechanisms [35, 36]. Notably, although TNIK-driven tumorigenesis has been predominantly linked to Wnt-dependent transcriptional programs, Wnt-independent oncogenic functions have been described in gastric cancer, lung cancer and prostate cancer [16, 37, 38], indicating that TNIK may engage alternative signaling networks depending on tumor context.

In contrast, in several other malignancies including colorectal, hepatocellular, pancreatic, and triple-negative breast cancers, TNIK has been implicated in tumor progression and is frequently discussed in the context of Wnt/β-catenin signaling, where it has been associated with enhanced stemness, invasion, and therapy resistance [15–19].

Conversely, in SHH-driven MB, our data suggest that TNIK-high tumors are associated with enrichment of Wnt-related transcriptional signatures and favorable clinical outcome, consistent with the reported antagonistic interaction between canonical Wnt signaling and SHH-driven tumorigenesis [33]. Taken together, these observations support the concept that both TNIK activity and Wnt signaling itself exert highly context-dependent biological effects, with their functional consequences determined by the dominant oncogenic signaling landscape of the respective tumor subtype.

Taken together, the divergent associations of TNIK expression across MB subtypes underscore its subtype-specific role in MB biology. In Group 3/4 tumors, elevated TNIK expression may indicate a more aggressive phenotype. In contrast, in SHH MBs, higher TNIK expression was associated with improved OS, as well as markers of Wnt pathway activity and lower proliferative indices at the transcriptomic level, suggesting a link to more favorable tumor biology. However, the association with improved survival was only partially reflected at TNIK protein levels, where a similar, however, non-significant trend was observed. These findings should therefore be interpreted with caution and considered hypothesis-generating.

Finally, TNIK may represent a potential therapeutic target in MB. Given the availability of TNIK inhibitors in other cancer entities, further studies are required to evaluate their relevance in MB. In this context, TNIK-high MB cell lines from different molecular backgrounds (e.g., UW228, DAOY, ONS76, and D458MED) may provide suitable in vitro models to investigate the functional consequences of TNIK inhibition or genetic ablation on tumor growth, survival, and treatment response in a subtype-specific manner, allowing further unraveling the subtype-specific functional properties of TNIK in MB.

## Supplementary Information

Below is the link to the electronic supplementary material.

Supplementary Material 1

## Data Availability

The data supporting the findings of this study are available from both public repositories and controlled institutional sources. Gene expression datasets analyzed in this study are publicly available from the Gene Expression Omnibus (GEO) under accession numbers *GSE85217*, *GSE21140*, and *GSE37418*. Proteomic datasets were obtained from the PRIDE database under accession numbers PXD048767, PXD006607, and PXD016832. FFPE tumor sections were provided by the Department of Pathology, Johns Hopkins University. These materials were used in accordance with institutional and national ethical standards. De-identified data generated in this study (including immunohistochemistry scoring and survival analyses) are available from the corresponding author upon reasonable request, subject to institutional approval and compliance with data-sharing policies.

## References

[1] Ramaswamy V, Remke M, Bouffet E et al (2016) Risk stratification of childhood medulloblastoma in the molecular era: the current consensus. Acta Neuropathol 131:821–831. 10.1007/s00401-016-1569-6 27040285 10.1007/s00401-016-1569-6 PMC4867119

[2] Northcott PA, Buchhalter I, Morrissy AS et al (2017) The whole-genome landscape of medulloblastoma subtypes. Nature 547:311–317. 10.1038/nature22973 28726821 10.1038/nature22973 PMC5905700

[3] Clifford SC, Meryl EL, Janet CL et al (2006) Wnt/Wingless Pathway Activation and Chromosome 6 Loss Characterise a Distinct Molecular Sub-Group of Medulloblastomas Associated with a Favourable Prognosis. Cell Cycle 5:2666–2670. 10.4161/cc.5.22.3446 17172831 10.4161/cc.5.22.3446

[4] Kool M, Korshunov A, Remke M et al (2012) Molecular subgroups of medulloblastoma: an international meta-analysis of transcriptome, genetic aberrations, and clinical data of WNT, SHH, Group 3, and Group 4 medulloblastomas. Acta Neuropathol 123:473–484. 10.1007/s00401-012-0958-8 22358457 10.1007/s00401-012-0958-8 PMC3306778

[5] Kool M, Jones DTW, Jäger N et al (2014) Genome Sequencing of SHH Medulloblastoma Predicts Genotype-Related Response to Smoothened Inhibition. Cancer Cell 25:393–405. 10.1016/j.ccr.2014.02.004 24651015 10.1016/j.ccr.2014.02.004 PMC4493053

[6] Taylor MD, Northcott PA, Korshunov A et al (2012) Molecular subgroups of medulloblastoma: the current consensus. Acta Neuropathol 123:465–472. 10.1007/s00401-011-0922-z 22134537 10.1007/s00401-011-0922-z PMC3306779

[7] Waszak SM, Northcott PA, Buchhalter I et al (2018) Spectrum and prevalence of genetic predisposition in medulloblastoma: a retrospective genetic study and prospective validation in a clinical trial cohort. Lancet Oncol 19:785–798. 10.1016/S1470-2045(18)30242-0 29753700 10.1016/S1470-2045(18)30242-0 PMC5984248

[8] Cavalli FMG, Remke M, Rampasek L et al (2017) Intertumoral Heterogeneity within Medulloblastoma Subgroups. Cancer Cell 31:737–754e6. https://doi.org/https://doi.org/10.1016/j.ccell.2017.05.005 28609654 10.1016/j.ccell.2017.05.005 PMC6163053

[9] Sharma T, Schwalbe EC, Williamson D et al (2019) Second-generation molecular subgrouping of medulloblastoma: an international meta-analysis of Group 3 and Group 4 subtypes. Acta Neuropathol 138:309–326. 10.1007/s00401-019-02020-0 31076851 10.1007/s00401-019-02020-0 PMC6660496

[10] Hovestadt V, Smith KS, Bihannic L et al (2019) Resolving medulloblastoma cellular architecture by single-cell genomics. Nature 572:74–79. 10.1038/s41586-019-1434-6 31341285 10.1038/s41586-019-1434-6 PMC6754173

[11] Pan Z, Bao J, Wei S (2025) Advancing medulloblastoma therapy: strategies and survival insights. Clin Exp Med 25:119. 10.1007/s10238-025-01648-5 40237916 10.1007/s10238-025-01648-5 PMC12003599

[12] Mahmoudi T, Li VSW, Ng SS et al (2009) The kinase TNIK is an essential activator of Wnt target genes. EMBO J 28:3329–3340. 10.1038/emboj.2009.285 19816403 10.1038/emboj.2009.285 PMC2776109

[13] Ewald CY, Pulous FE, Lok SWY et al (2024) TNIK’s emerging role in cancer, metabolism, and age-related diseases. Trends Pharmacol Sci 45:478–489. 10.1016/j.tips.2024.04.010 38777670 10.1016/j.tips.2024.04.010

[14] Shitashige M, Satow R, Jigami T et al (2010) Traf2- and Nck-Interacting Kinase Is Essential for Wnt Signaling and Colorectal Cancer Growth. Cancer Res 70:5024–5033. 10.1158/0008-5472.CAN-10-0306 20530691 10.1158/0008-5472.CAN-10-0306

[15] Masuda M, Uno Y, Ohbayashi N et al (2016) TNIK inhibition abrogates colorectal cancer stemness. Nat Commun 7:12586. 10.1038/ncomms12586 27562646 10.1038/ncomms12586 PMC5007443

[16] Yu D-H, Zhang X, Wang H et al (2014) Erratum: The essential role of TNIK gene amplification in gastric cancer growth. Oncogenesis 3:e93–e93. 10.1038/oncsis.2014.9 24637493 10.1038/oncsis.2014.9 PMC4038394

[17] Jin J, Jung HY, Wang Y et al (2014) Nuclear expression of phosphorylated TRAF2- and NCK-interacting kinase in hepatocellular carcinoma is associated with poor prognosis. Pathol Res Pract 210:621–627. 10.1016/j.prp.2013.10.007 25160513 10.1016/j.prp.2013.10.007

[18] Zhang Y, Jiang H, Qin M et al (2016) TNIK serves as a novel biomarker associated with poor prognosis in patients with pancreatic cancer. Tumor Biology 37:1035–1040. 10.1007/s13277-015-3881-5 26269113 10.1007/s13277-015-3881-5

[19] Sato K, Padgaonkar AA, Baker SJ et al (2021) Simultaneous CK2/TNIK/DYRK1 inhibition by 108600 suppresses triple negative breast cancer stem cells and chemotherapy-resistant disease. Nat Commun 12:4671. 10.1038/s41467-021-24878-z 34344863 10.1038/s41467-021-24878-z PMC8333338

[20] Poma AM, Torregrossa L, Bruno R et al (2018) Hippo pathway affects survival of cancer patients: extensive analysis of TCGA data and review of literature. Sci Rep 8:10623. 10.1038/s41598-018-28928-3 30006603 10.1038/s41598-018-28928-3 PMC6045671

[21] Camp RL, Dolled-Filhart M, Rimm DL (2004) X-tile: a new bio-informatics tool for biomarker assessment and outcome-based cut-point optimization. Clin Cancer Res 10:7252–7259. 10.1158/1078-0432.CCR-04-0713 15534099 10.1158/1078-0432.CCR-04-0713

[22] Barretina J, Caponigro G, Stransky N et al (2012) The Cancer Cell Line Encyclopedia enables predictive modelling of anticancer drug sensitivity. Nature 483:603–607. 10.1038/nature11003 22460905 10.1038/nature11003 PMC3320027

[23] Shih DJH, Northcott PA, Remke M et al (2014) Cytogenetic Prognostication Within Medulloblastoma Subgroups. J Clin Oncol 32:886–896. 10.1200/JCO.2013.50.9539 24493713 10.1200/JCO.2013.50.9539 PMC3948094

[24] Goschzik T, zur Muehlen A, Doerner E et al (2021) Medulloblastoma in Adults: Cytogenetic Phenotypes Identify Prognostic Subgroups. J Neuropathol Exp Neurol 80:419–430. 10.1093/jnen/nlab020 33870422 10.1093/jnen/nlab020

[25] Ray S, Chaturvedi NK, Bhakat KK et al (2022) Subgroup-specific diagnostic, prognostic, and predictive markers influencing pediatric medulloblastoma treatment. Diagnostics 12 10.3390/diagnostics12010061 PMC8774967 35054230

[26] Uhlén M, Fagerberg L, Hallström BM et al (2015) Tissue-based map of the human proteome. Science (1979) 347:1260419. 10.1126/science.1260419 10.1126/science.1260419 25613900

[27] Uhlén M, Björling E, Agaton C et al (2005) A Human Protein Atlas for Normal and Cancer Tissues Based on Antibody Proteomics. Mol Cell Proteom 4:1920–1932. 10.1074/mcp.M500279-MCP200 10.1074/mcp.M500279-MCP200 16127175

[28] Schürch C, Riether C, Matter MS et al (2012) CD27 signaling on chronic myelogenous leukemia stem cells activates Wnt target genes and promotes disease progression. J Clin Invest 122:624–638. 10.1172/JCI45977 22232214 10.1172/JCI45977 PMC3266773

[29] Yang Y-F, Yu B, Zhang X-X, Zhu Y-H (2021) Identification of TNIK as a novel potential drug target in thyroid cancer based on protein druggability prediction. Medicine 100:e25541. 10.1097/MD.0000000000025541 33879700 10.1097/MD.0000000000025541 PMC8078263

[30] Weber LI, Timpen LE, Egger-Hörschinger A-S et al (2026) Reactivation of the silenced *BASP1* gene suppresses oncogenic WNT signaling in human colorectal cancer cells. Proc Natl Acad Sci 123. 10.1073/pnas.2524159123 10.1073/pnas.2524159123 PMC12974518 41785318

[31] Zhang T-T, Yi W, Dong D-Z et al (2024) METTL3-mediated upregulation of FAM135B promotes EMT of esophageal squamous cell carcinoma via regulating the Wnt/β-catenin pathway. Am J Physiology-Cell Physiol 327:C329–C340. 10.1152/ajpcell.00529.2023 10.1152/ajpcell.00529.2023 38881420

[32] Wu X, Zhong Y, Zhang H, Li M (2024) MiR-5590-3p inhibits the proliferation and invasion of ovarian cancer cells through mediating the Wnt/β-catenin signaling pathway by targeting TNIK. Histol Histopathol 39:345–355. 10.14670/HH-18-636 37318197 10.14670/HH-18-636

[33] Pöschl J, Bartels M, Ohli J et al (2014) Wnt/β-catenin signaling inhibits the Shh pathway and impairs tumor growth in Shh-dependent medulloblastoma. Acta Neuropathol 127:605–607. 10.1007/s00401-014-1258-2 24531885 10.1007/s00401-014-1258-2

[34] Coco S, Valdora F, Bonassi S et al (2011) Chromosome 9q and 16q Loss Identified by Genome-Wide Pooled-Analysis Are Associated with Tumor Aggressiveness in Patients with Classic Medulloblastoma. OMICS 15:273–280. 10.1089/omi.2010.0103 21348762 10.1089/omi.2010.0103

[35] Manoranjan B, Venugopal C, Bakhshinyan D et al (2020) Wnt activation as a therapeutic strategy in medulloblastoma. Nat Commun 11:4323. 10.1038/s41467-020-17953-4 32859895 10.1038/s41467-020-17953-4 PMC7455709

[36] Northcott PA, Korshunov A, Witt H et al (2010) Medulloblastoma Comprises Four Distinct Molecular Variants. J Clin Oncol 29:1408–1414. 10.1200/JCO.2009.27.4324 20823417 10.1200/JCO.2009.27.4324 PMC4874239

[37] Torres-Ayuso P, An E, Nyswaner KM et al (2021) TNIK Is a Therapeutic Target in Lung Squamous Cell Carcinoma and Regulates FAK Activation through Merlin. Cancer Discov 11:1411–1423. 10.1158/2159-8290.CD-20-0797 33495197 10.1158/2159-8290.CD-20-0797 PMC8178189

[38] Guo J, Liang J, Wang Y et al (2024) TNIK drives castration-resistant prostate cancer via phosphorylating EGFR. iScience 27:108713. 10.1016/j.isci.2023.108713 38226156 10.1016/j.isci.2023.108713 PMC10788198

