# Supplementary material for: Subtype-specific prognostic impact of TNIK in medulloblastoma
Source: J Neurooncol. 2026 May 8;177(3):140. doi: 10.1007/s11060-026-05554-y (PMC13156172; doi:10.1007/s11060-026-05554-y)
Supplement: Supplementary file 1 — Supplementary Material 1 [file 11060_2026_5554_MOESM1_ESM.docx]

Supplemental Data

Subtype-Specific Prognostic Impact of TNIK in Medulloblastoma

Franz-Leonard Klaus^1^, Theoni Maragkou^2^, Claire Delbridge^1^, Charles G Eberhardt^3^, Ekkehard Hewer^4^, Antonia Gocke^5,6^, Ramin Radpour^7,8^, Christian Mawrin^9^, Carolin Mogler^1^, Julia E Neumann^5,10^, Stefan Forster^1†^

1 Institute of Pathology, Technical University Munich, Munich, Germany

2 Institute of Tissue Medicine and Pathology, University of Bern, Bern, Switzerland

3 Department of Pathology, School of Medicine, Johns Hopkins University, Baltimore, MD, USA

4 Department of Laboratory Medicine and Pathology, Institute of Pathology, Lausanne University Hospital and University of Lausanne, Lausanne, Switzerland

5 Center for Molecular Neurobiology Hamburg (ZMNH), University Medical Center Hamburg-Eppendorf (UKE), Hamburg, Germany

6 Center for Diagnostics: Section Mass Spectrometry and Proteomics, University Medical Center Hamburg-Eppendorf, Hamburg, Germany

7 Tumor Immunology, Department for BioMedical Research (DBMR), University of Bern, Bern, Switzerland.

8 Department of Medical Oncology, Inselspital, Bern University Hospital, University of Bern, Bern, Switzerland.

9 Department of Neuropathology, Otto-von-Guericke-University, Magdeburg, Germany.

10 Institute of Neuropathology, University Medical Center Hamburg-Eppendorf (UKE), Hamburg, Germany

†Correspondence: Institute of Pathology, Technical University Munich, Munich, Germany; e-mail: stefan.forster@tum.de

**Materials & Methods**

**Immunohistochemistry Staining**

Formalin-fixed and paraffin-embedded (FFPE) unstained tissue microarray sections were obtained from MB patients with informed consent and were provided by Department of Pathology, School of Medicine, Johns Hopkins University, Baltimore. The TMA comprised a total of 53 primary medulloblastoma samples, including SHH (n = 24), Group 3 (n = 5), Group 4 (n = 10) and n = 14 tumors with unknown molecular background. Stainings for Hematoxylin and Eosin (H&E) and TNIK (sc-377215; Santa Cruz) were conducted using a Leica BOND RX automated immunostainer (Leica Biosystems). The specificity of TNIK immunostaining was validated using a CRISPR/Cas9-mediated TNIK knockout cell line (EBC-1), which confirmed loss of TNIK immunoreactivity and confirmed antibody specificity. Stained TMA sections were independently evaluated by two pathologists in training (SF and FLK) who were blinded to clinical and molecular data. TNIK expression was assessed based on staining intensity. Staining intensity was scored using a four-tier scale: 0 = no detectable staining, 1 = weak staining, 2 = moderate staining and 3 = strong staining. In cases of discrepant scoring, a consensus score was established following joint review together with a FMH board-certified neuropathologist (TM).  All slides were scanned and digitally evaluated using the Pannoramic P250 Flash III digital slide scanner and SlideViewer software (3DHistech).

**Mass Spectrometry Analysis**

Proteomic data from publicly available datasets (*PXD048767*, *PXD006607*, *PXD016832*) were accessed through the PRIDE database and integrated using R (version 4.3.2) using the BERT algorithm (version 1.5.0)^23–26^. Patients were stratified into TNIK high and TNIK low utilizing the median TNIK abundance as a cut-off. Kaplan-Meier survival analysis, log-rank testing and visualizations were performed in R (v.4.2.0) using the packages survival, survminer, ggsurvfit, ggplot2 and ggbeeswarm.

**Statistical Analysis**

Statistical analyses were performed using GraphPad Prism v8 (GraphPad Software, USA). Tests included Student’s t-test, one-way ANOVA with Tukey HSD post-hoc test, and Kaplan–Meier survival analysis with log-rank testing. Survival cutoffs were determined using X-Tile software based on the mean expression of candidate genes. False discovery rate (FDR) correction (Benjamini–Hochberg) was applied to transcriptomic analysis datasets. Data are presented as mean ± SD, and *P* < 0.05 was considered statistically significant. Additional test details are provided in the figure legends.


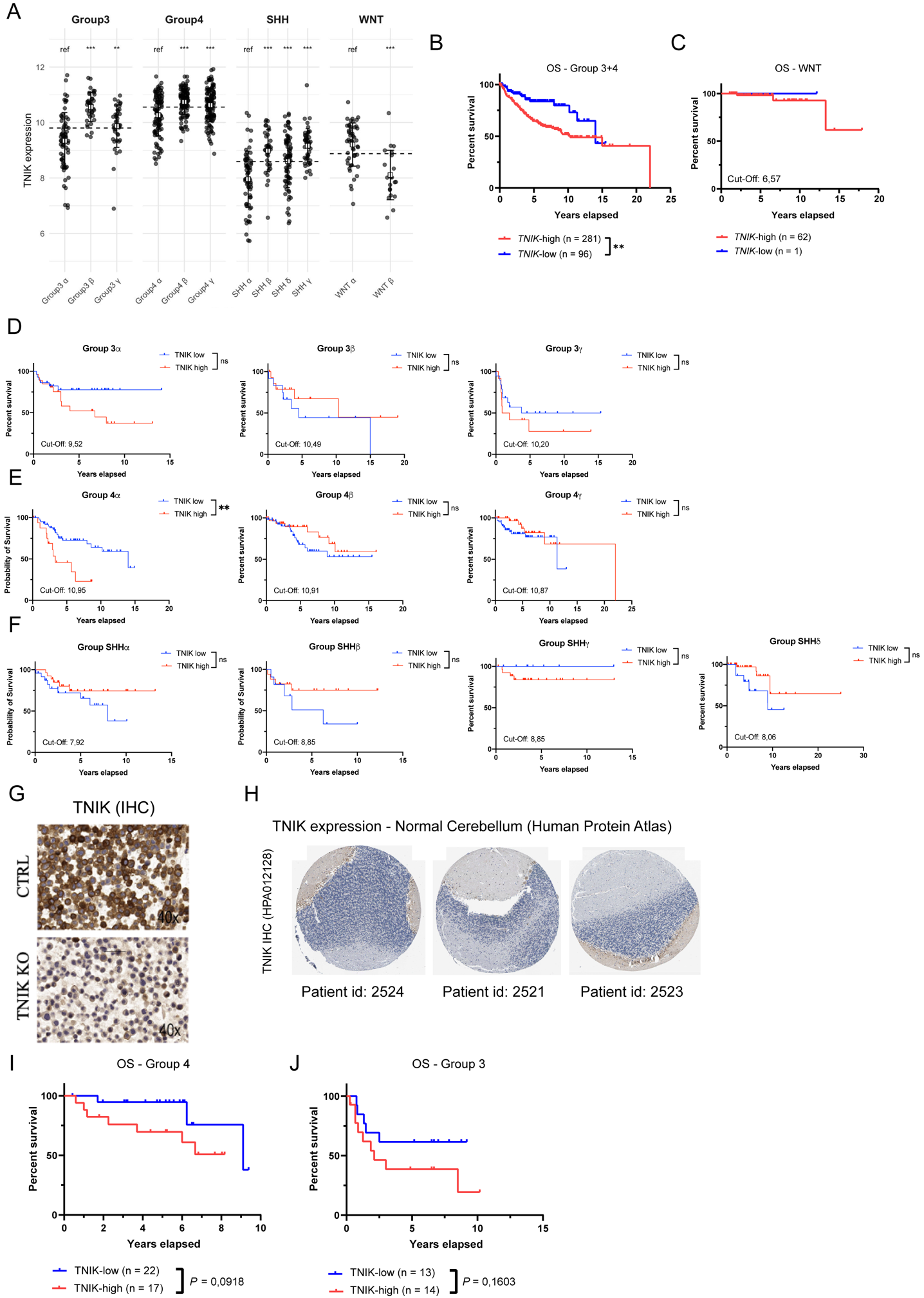


**Supplemental Figure 1:** TNIK mRNA expression levels across MB subtypes (A). Kaplan-Meier survival curve of combined *TNIK*-high and *TNIK*-low Group 3 and Group 4 MBs (B). Kaplan-Meier survival curve of *TNIK*-high and *TNIK*-low Wnt-activated MBs (C). Kaplan-Meier survival curves across MB subtypes within Group 3 (D), Group 4 (E) and SHH (F). TNIK antibody specificity was confirmed in a TNIK knock-out cell line (EBC-1); pictures were taken at 40x magnification (G). TNIK expression analyzed in (n = 3) normal cerebellum, data extracted from the human protein atlas (<https://www.proteinatlas.org/>) (H). Overall survival of Group 4 (I) and Group 3 MBs (J) stratified into TNIK-low and TNIK-high subgroups based on the median mass spectrometry cutoff. Statistics: Two-sided Mann-Whitney U tests (A), log-rank test (B-F, I and J). **, *P* < 0.01; ***, *P* < 0.001; Data are shown as mean with SD.


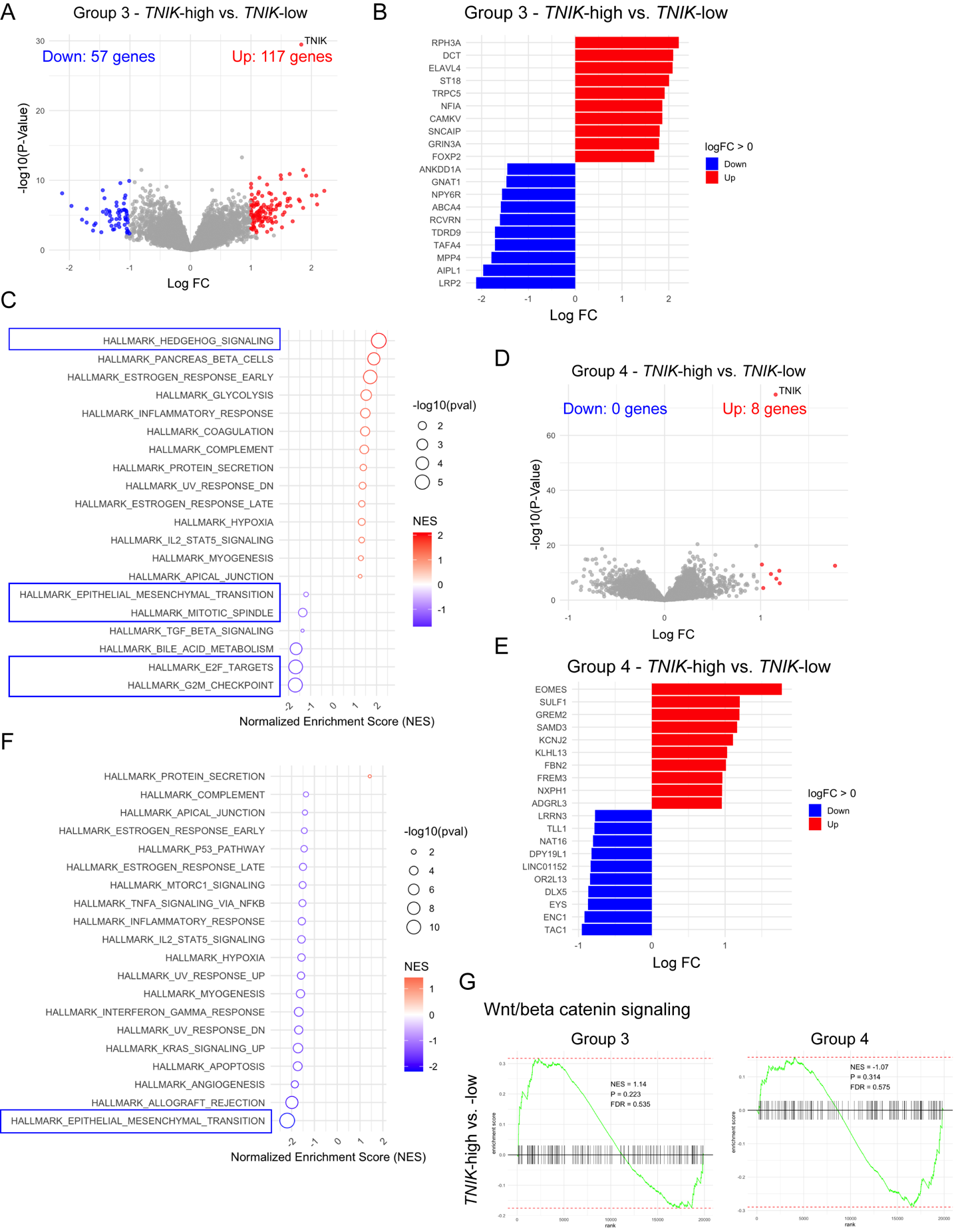


**Supplemental Figure 2:** Volcano plot visualizing differentially expressed genes in *TNIK*-high versus *TNIK*-low Group 3 MB patients (A). Top ten upregulated and downregulated genes in *TNIK*-high Group 3 MBs (B). Gene set enrichment analyses (GSEA) of *TNIK*-high versus *TNIK*-low Group 3 MBs (C). Volcano plot visualizing differentially expressed genes in *TNIK*-high versus *TNIK*-low Group 4 MB patients (D). Top ten upregulated and downregulated genes in *TNIK*-high Group 4 MBs (E). GSEA of *TNIK*-high versus *TNIK*-low Group 4 MBs (F). Enrichment of genes involved in Wnt/β-catenin signaling in *TNIK*-high versus *TNIK*-low MB patients across Group 3 and Group 4 subgroups (G).
